# Annual shifts of flow regime alteration: new insights from the Chaishitan Reservoir in China

**DOI:** 10.1038/s41598-018-19717-z

**Published:** 2018-01-23

**Authors:** Yongyong Zhang, Xiaoyan Zhai, Tongtiegang Zhao

**Affiliations:** 1 Key Laboratory of Water Cycle and Related Land Surface Processes, Institute of Geographic Sciences and Natural Resources Research, Chinese Academy of Sciences, Beijing, 100101 China; 2 China Institute of Water Resources and Hydropower Research, Beijing 100038, China; Research Center on Flood and Drought Disaster Reduction of the Ministry of Water Resources, Beijing, 100038 China; 3 Department of Infrastructure Engineering, University of Melbourne, Parkville, Victoria, 3052 Australia

## Abstract

Reservoir regulation is variable for flow regime alterations and mainly depends on operational objectives and hydro-meteorological conditions. In this study, the flow regime metrics (i.e., magnitude, variability and frequency, duration, timing and rate of change) are adopted to describe variations in both long-term inflow and outflow series of the Chaishitan Reservoir in China. Deviations between the inflow and outflow metrics are calculated to assess the flow regime alterations at annual scale. Further, dimensions of both time and flow regimes are reduced by multivariate statistical analysis, and the regulation patterns and their annual shifts are identified. Results show that: four regulation patterns are identified from 2004 to 2015. The regulation is gradually enhanced over time with typical features of different hydrological years. In dry years, the pattern is slightly regulated flow regimes with slightly discharging stored water and flattening outflow, moderate stability and intermittency. In normal years, the pattern is slightly regulated flow regimes with extremely increasing flow magnitude in the pre-nonflood season, high stability and slight intermittency. In wet years, the pattern is moderately regulated flow regimes with moderately decreasing flow magnitude in the flood season but extremely increasing flow magnitude in the nonflood season, slight stability and high intermittency.

## Introduction

Natural flow regime alteration caused by reservoir regulations is a prominent issue in global water system projects^1,2^ and can deteriorate water environment and riverine ecology^3–5^. Over half of the 292 global large river systems are affected by dams in terms of flow regulation and channel fragmentation^6^. River fragments have increased by 801% via dam construction in the USA^7^. As a leading country of dam construction, China has built approximately 86 thousand dams, which accounted for over half of the world’s total by the end of 2007^2^. Recently, concerns have been raised regarding the effects of global dam construction and regulation on natural water cycle in terms of river geomorphology changes/connectivity disruption and sediment budgets^8,9^, hydrological variability reduction^10,11^, surface water pollution and eutrophication^12,13^, and biotic diversity degradation^14,15^, among others. Therefore, it is urgent and critical to quantify the impact of dam regulation on flow regimes to provide scientific support to optimize the regulation rules of dams.

Currently, the focus of dam regulation assessments is on hydrograph changes based on making a comparison between the inflow and outflow of a dam (inflow-outflow differences assessment)^16,17^ or the flows between pre- and post- dam periods (pre-post dam flow differences assessment)^18–21^. The assessment of inflow-outflow differences is restricted to long-term synchronous observations of both inflow and outflow, while the pre-post dam flow differences assessment is limited by the same external restrictions (e.g., climate, underlying surface) between pre-dam and post-dam periods if the observations are used for the assessment or by model performance if mathematical models are adopted to generate the pre-post dam flow series^22^. In the meantime, the hydrograph changes are usually described by some flow related metrics, such as water resource metrics (e.g., flow magnitude)^16,23^, eco-flow regime metrics (e.g., magnitude, frequency and variability, duration, timing and rating of flow events)^18,24^, or comprehensive indices, e.g., combined river impact factor^25^, flow fragmentation indices^26,27^ and regulation indices^27,28^. The water resource metrics only focus on the one-sided aspect of the hydrograph, while the comprehensive indices are so general that not all flow signatures can be captured in detail. Although the eco-flow regime metrics are considered to be time-consuming and highly dimensional^25^, they can capture all hydrograph changes. Moreover, multivariate statistical analysis methods are widely adopted to identify the representative characteristics from numerous pieces of flow regime metrics and without losing metric information^29^. Some typical methods include Analysis of Similarity, principal component analysis, cluster analysis and set pair analysis^22,30^.

This paper hypothesizes that the impact patterns of reservoir regulations on flow regime characteristics are different across different years. This hypothesis is based on the fact that climate variability, upstream inflow dynamics and operational objectives, as well as reservoir regulation rules, are time-varying^31,32^. Note that most previous flow regime assessments have mainly focused on the annual average steady-state variations of flow regimes and ignored their temporal shifts^6,28,33,34^. The reservoir regulation usually belongs to a certain pattern, such as “run-of-river” for flood control, “storage” for water supply, “peaking” for hydroelectric generation^10,33^, and so forth. Many studies have been implemented to investigate the regulation patterns at the basin^35^, continental^10,20^ and global^27^ scales. The temporal shifts of dam regulation are still poorly understood, which can cause understanding deviations in the dam regulation impacts, and restrict scientific dam regulations.

**Figure 1:**
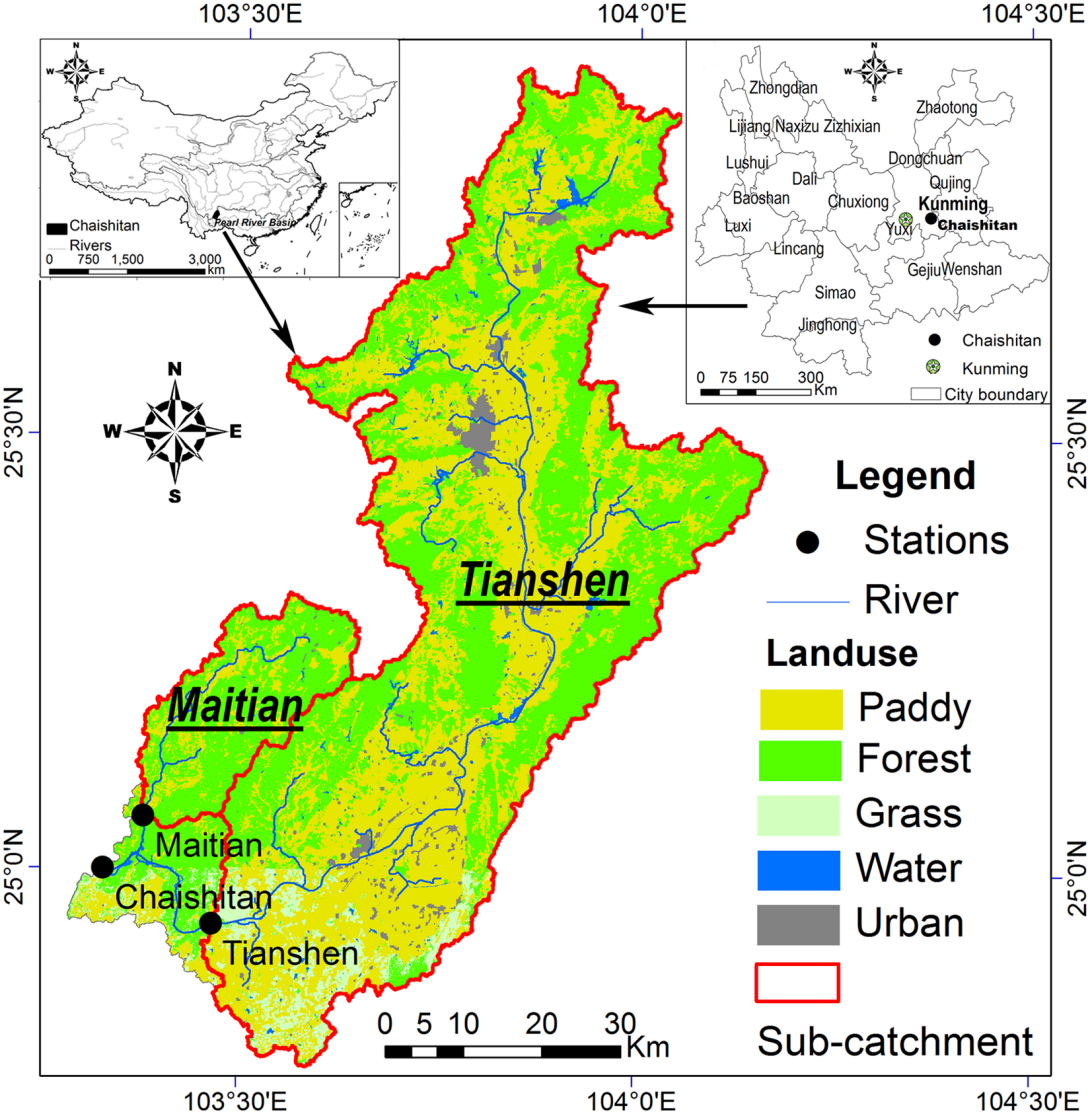
Locations of the Chaishitan catchment, main land use areas, and inflow and outflow stations. This figure was generated using ArcGIS version 10.0 (http://desktop.arcgis.com/en/).

Therefore, this paper elaborates on the annual shifts of flow regime alteration by reservoir regulation. The investigation focuses on the Chaishitan Reservoir (Fig. 1), which is the leading reservoir of the upstream cascade development of the Pearl River and is one of the major water projects of the 13th Five-Year Plan of China (2016–2020). This reservoir plays a critical role in protecting the environment of plateau lakes and controlling stony desertification, which are of great significances for the socio-economic development and ecological construction of the upstream provinces of the Pearl River Basin. The principal objectives of this study are to: (1) describe the long-term flow regime characteristics of both reservoir inflow and outflow in terms of the magnitude, frequency, variation, duration, timing, and rate of changes; (2) assess their inter-annual variations based on the deviation of synchronous inflow and outflow regime characteristics; and (3) identify representative impact patterns of reservoir regulation and their annual shifts by dimensionality reduction approaches (e.g. principal component analysis and cluster analysis).

## Results

### Annual impact variations for flow regime metrics

**Figure 2:**
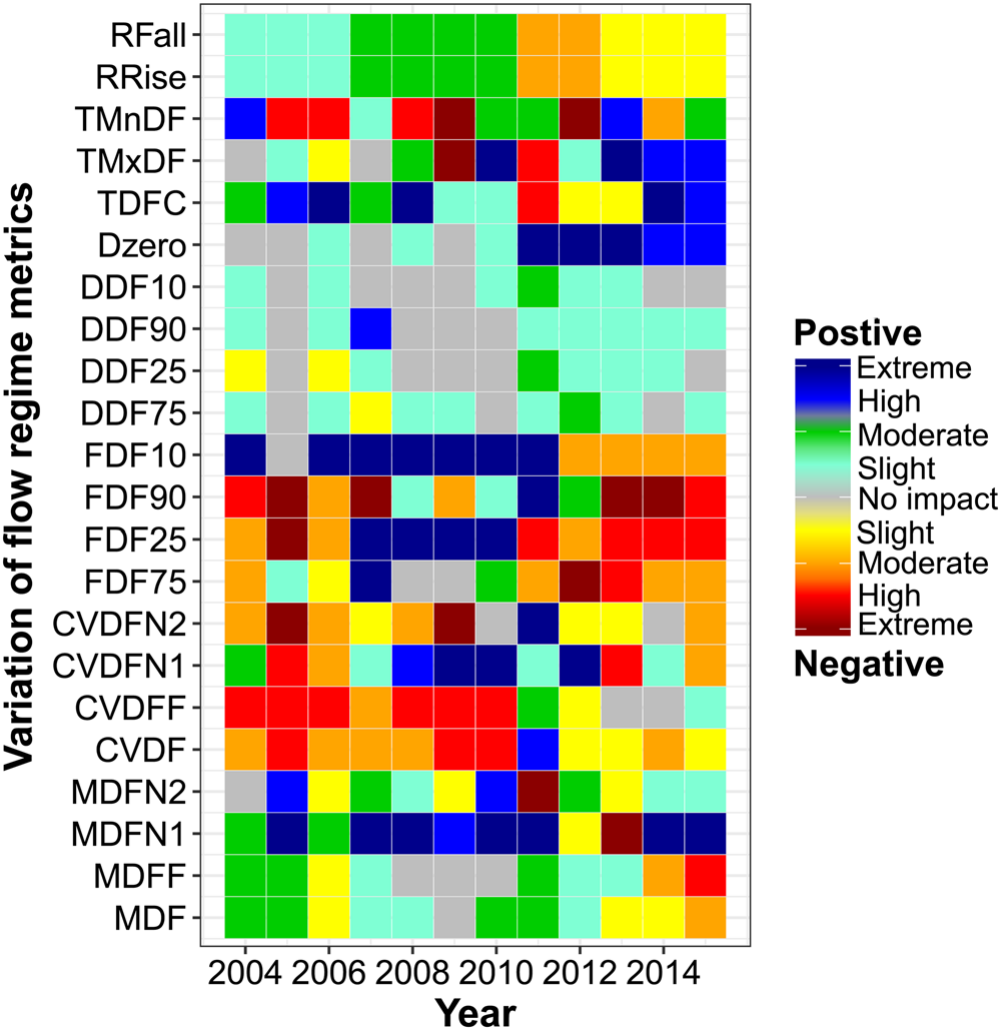
Heat map showing the impact grades of the Chaishitan Reservoir on the flow regime metrics according to the changes of individual metrics between inflow and outflow. This figure was generated using the R platform (version 3.1.1)^40^ and the ggplot function in the ggplot2 package (version 2.2.0)^44^.

For the flow magnitude, the annual impact variations of both MDF (mean daily runoff) and MDFF (mean daily runoff in the flood season from April to September) are not beyond the moderate grades and changed from the positive to negative grades around 2013 (Fig. 2). Most annual MDFN1s (mean daily runoff in the pre-nonflood season from January to March) are positively impacted, particularly in 2005, 2007~2011, 2014 and 2015 (the extreme grades), while the impact variations of MDFN2 (mean daily runoff in the post-nonflood season from October to December) are not obviously consistent over time, and are concentrated at the slight negative or positive grades.

For the flow variability and frequency, the impacts of all the flow variability metrics are negative in most years, except for the positive impacts of CVDFN1 (Coefficient of Variation of mean daily runoff in the pre-nonflood season) in 2008~2012 and 2014, CVDFN2 (Coefficient of Variation of mean daily runoff in the post-nonflood season) in 2011 and CVDFF (Coefficient of Variation of mean daily runoff in the flood season) in 2014. Most annual impacts on the low flow frequencies are negative, mainly in 2004~2006 and 2011~2015 for FDF75 (low flow spell count: 75th percentile of MDF), and in 2004~2007 and 2013~2015 for FDF90 (extreme low flow spell count: 90th percentile of MDF). The annual impacts on high flow frequencies vary from positively extreme to negatively moderate or slight grades around 2011, particularly for FDF10 (extreme high flow spell count: 10th percentile of MDF).

For the flow duration, the annual impact variations of extreme low and high flow events (DDF90 and DDF10), and low and high flow events (DDF75 and DDF25) are at slightly positive grades from 2004 to 2007 and from 2011 to 2014, while those in the other years have no impacts. However, the impacts on Dzero (number of zero-flow days) are changed from no impact to extremely positive grades. For the flow timing, the impacts on both TMnDF (Julian date of annual minimum daily runoff) and TMxDF (Julian date of annual maximum daily runoff) changed from negative grades to positive grades approximately 2010, particularly for TMnDF. For the flow ratings of changes, the annual impact variations of both RRise (mean rate of positive changes in flow from one day to the next) and RFall (mean rate of negative changes in flow from one day to the next) are consistent over time, and are divided into four parts, i.e., slightly positive grade from 2004 to 2006, moderately positive grade from 2007 to 2010, moderately negative grade from 2011 to 2012 and slightly negative grade from 2013 to 2015.

Therefore, the regulation of the Chaishitan Reservoir alternately decreases or increases the mean daily runoff in individual years and the flood seasons at the slight or moderate grade over time, and increases obviously in the nonflood seasons, particularly in the pre-nonflood seasons; as a result, the regulation decreases flow variability except in the pre-nonflood seasons, and it decreases the frequencies of low flow events whereas it increases the frequencies of high flow events, particularly for extreme high flow events. It also extends the flow event durations, increases the mean rates of both positive and negative changes in flow before 2011 and then turns to decrease the rates. However, the impacts on flow timing are without obvious rules during the study period. The most impactful metrics are MDFN1 in flow magnitude, FDF25 and FDF10 in flow frequency, Dzero in flow duration and TDFC (Colwell’s Constancy of mean daily runoff) in flow timing.

### Impact pattern classifications

**Figure 3:**
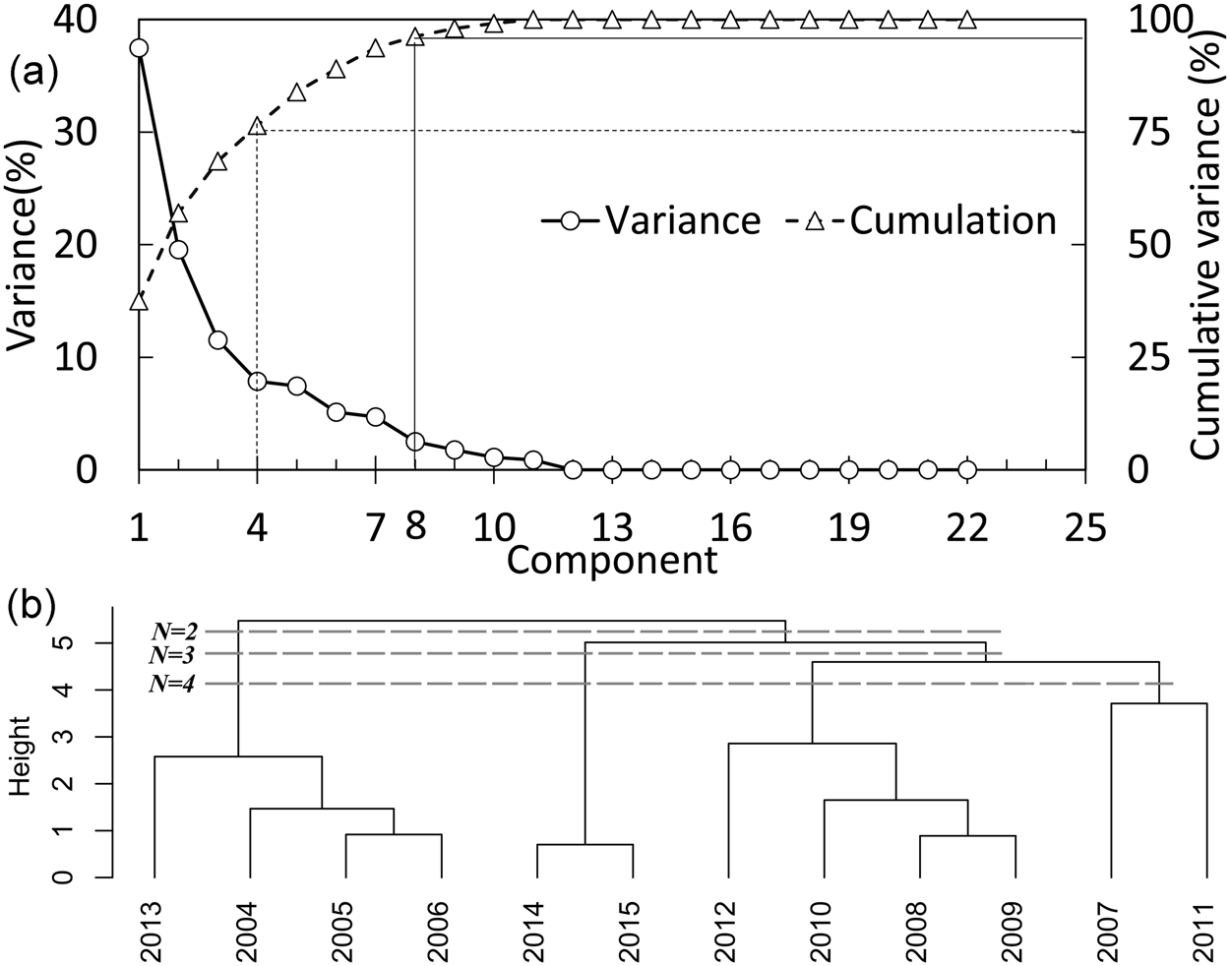
Scree plot (**a**) of the variances of the individual principal components and their cumulative variances, and hierarchical diagram (**b**) for impact classification of the Chaishitan Reservoir by cluster analysis when four principal components are selected. The scree plot was generated using Microsoft Office Excel 2013, and the hierarchical diagram was generated using the R platform (version 3.1.1)^40^ and the hcluster function in the amap package^41^.

The impact grade series of all flow regime metrics can be composited into four, five, six, seven and eight independent principal components, which explain 76.45%, 83.90%, 89.30%, 93.73% and 96.24%, respectively, of the entire impact grade series (Fig. 3a). All the component compositions are individually adopted for cluster analysis; the cluster performance results are shown in Fig. 3b and Table 1. The four-cluster classification for the four principal components is the most robust with the largest Goodman–Kruskal index (*GKI*: 0.76) and the smallest C index (*CI*: 0.10). All of the class sizes are also no less than two.

**Table 1:** Cluster performance assessment for different principal components.

principal components	Clusters	*GKI*	*CI*	Minimum size
4 principal components with cumulative variance of 76.45%	2	0.15	0.39	4
	3	0.33	0.30	2
	**4**	**0.76**	**0.10**	**2**
5 principal components with cumulative variance of 83.90%	2	0.23	0.37	5
	3	0.14	0.40	2
	4	0.48	0.23	2
6 principal components with cumulative variance of 89.30%	2	0.23	0.32	4
	3	0.32	0.30	2
	4	0.44	0.27	2
7 principal components with cumulative variance of 93.73%	2	−0.02	0.45	5
	3	0.00	0.45	2
	4	0.35	0.31	2
8 principal components with cumulative variance of 96.24%	2	−0.12	0.47	3
	3	−0.25	0.57	2
	4	−0.46	0.62	2

**Figure 4:**
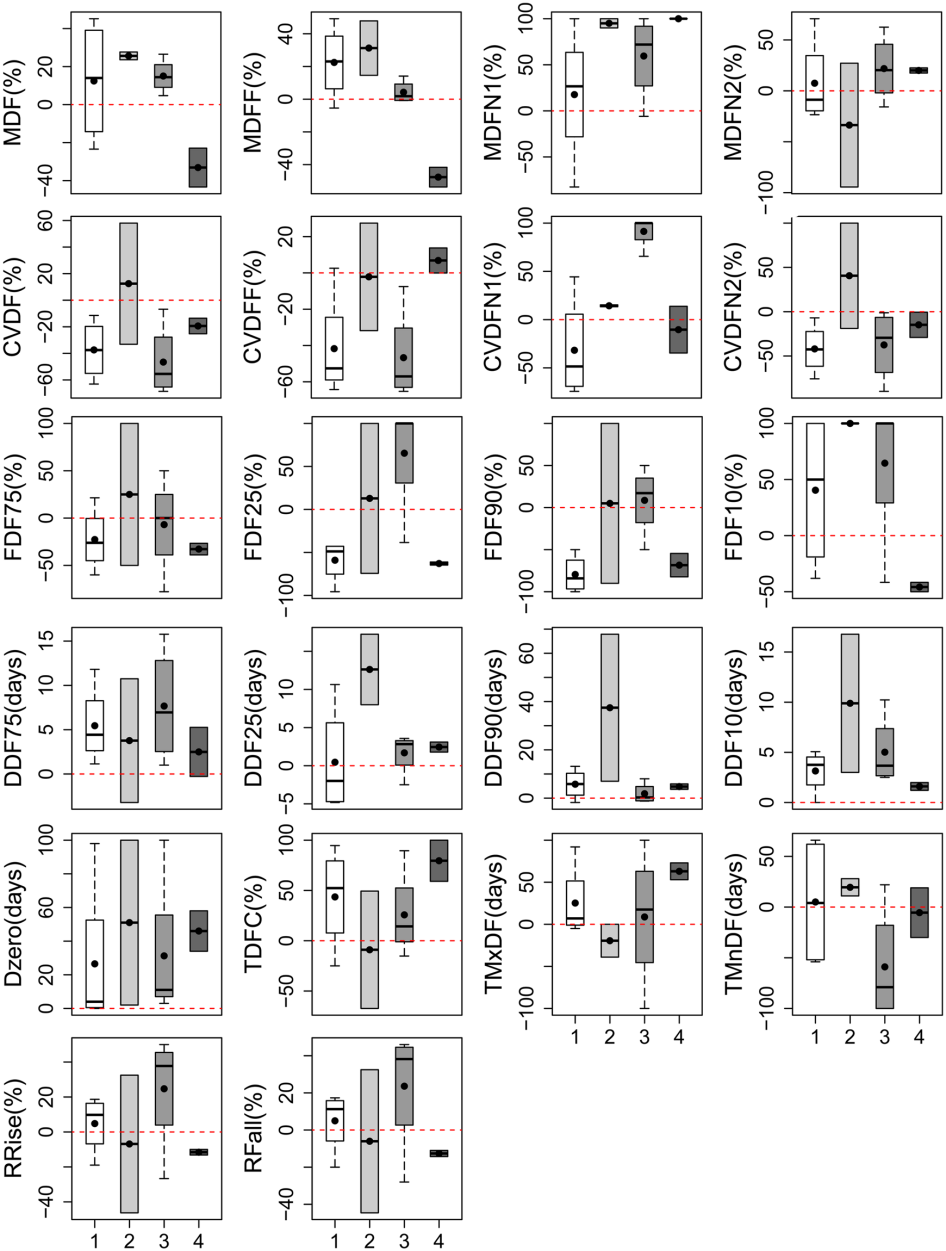
Boxplot for the variations of the flow regime metrics for each class. Boxes and bands define the 25^th^, median and 75^th^ percentile values, and the vertical bars (whiskers) define the 10^th^ and 90^th^ percentile values. The mean values are defined by the black solid dot symbols. This figure was generated using the R platform (version 3.1.1)^40^ and the boxplot function in the boxplotdbl package (version 1.2.2)^45^.

The annual variations in flow regime in four years (2004, 2005, 2006 and 2013) belong to Class 1, accounting for 33.3% of the total period. The regulation of the Chaishitan Reservoir slightly increases the mean daily flow magnitude in the flood season, the nonflood season and the whole year. For the flow variability and frequency, the regulation moderately decreases the Coefficient of Variation (CV) of the mean daily flow in the flood season, the nonflood season and the whole year, and it decreases the frequencies of both low and high flow events, except FDF10 (Fig. 4). Moreover, the regulation slightly increases the durations of all flow events (except DDF25), moderately increases the zero-flow days, delays the Julian dates of annual maximum daily flows, and slightly increases the mean rates of both positive and negative changes in flow events. Thus, the impact pattern of the Chaishitan Reservoir regulation in Class 1 is characterized as Slightly Regulated Flow regimes with Slight Increasing flow Magnitude, Moderate Stability and Moderate Intermittency (SRF:SIM-MS-MI).

The annual variations of flow regime in two years (2007 and 2011) belong to Class 2, accounting for only 16.7% of the total period. The regulation of the Chaishitan Reservoir slightly increases the mean daily flow magnitude; the regulation moderately increases the mean daily flow in the flood season and extremely increases in the pre-nonflood season, whereas it moderately decreases in the post-nonflood season. The regulation slightly increases the CV of the mean daily flow; the regulation increases the CV of mean daily flow slightly in the pre-nonflood season and moderately in the post-nonflood season with no obvious changes in the flood season. The regulation also increases the frequencies of high and low flow events, (particularly for the extremely high flow events), slightly extends the flow durations, highly increases the zero-flow days, and moderately advances the Julian dates of annual maximum daily flows, whereas it delays the Julian dates of the annual minimum and slightly decreases the mean rates of both positive and negative changes in flow events. Thus, the impact pattern in Class 2 is characterized as Slightly Regulated Flow regimes with Moderately Increasing flow Magnitude except the post-nonflood season, Slight Variability and High Intermittency (SRF:MIM-SV-HI).

The annual variations of flow regime in four years (2008, 2009, 2010 and 2012) belong to Class 3, accounting for 33.3% of the total period. The regulation of the Chaishitan Reservoir slightly increases the mean daily flow magnitude; the regulation extremely increases the mean daily flows in the pre-nonflood season and slightly increases them in the post-nonflood season, with no obvious changes in the flood season. The regulation highly decreases the CV of mean daily flow in the whole year and the flood season, and slightly decreases it in the post-nonflood season, whereas it extremely increases the CV in the pre-nonflood season, highly increases the frequencies of high flow events, and only slightly increases the frequencies of extremely low flow events. Moreover, the regulation slightly extends the flow durations of low flow events and extremely high flow events, has no obvious impacts on those of extremely low flow events and high flow events, moderately increases the zero-flow days, slightly delays the Julian dates of annual maximum and minimum daily flows, and moderately increases the mean rates of both positive and negative changes in flow events. Thus, the impact pattern in Class 3 is characterized as Slightly Regulated Flow regimes with Extremely Increasing flow Magnitude in the pre-nonflood season, High Stability and Slight Intermittency (SRF:EIMN-HS-MI).

The annual variations of flow regime in two years (2014 and 2015) belong to Class 4, accounting for only 16.7% of the total period. The regulation of the Chaishitan Reservoir moderately decreases the mean daily flow magnitude in the whole year and the flood season, and extremely decreases in the pre-nonflood season and slightly in the post-nonflood season. The regulation slightly decreases the CV of mean daily flow in the whole year and nonflood season (both pre- and post-), whereas it slightly increases in the flood season, moderately decreases the frequencies of low flow events and extremely high flow events, and highly decreases the frequencies of high flow events and extremely low flow events. Moreover, the regulation slightly extends the flow durations, but highly increases the zero-flow days, highly delays the Julian dates of annual maximum daily flows, and slightly decreases the mean rates of both positive and negative changes in flow events. Thus, the impact pattern in Class 4 is characterized as Moderately Regulated Flow regimes with Moderately Decreasing flow Magnitude in the flood season, Slight Stability and High Intermittency (MRF:MDM-SS-HI).

### Annual shifts of the impact patterns

**Figure 5:**
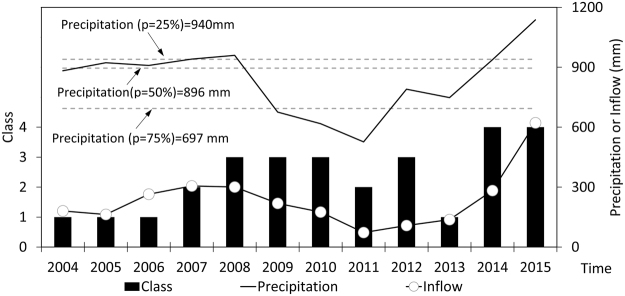
Annual shifts of the impact patterns of the Chaishitan Reservoir regulation, the upstream precipitation and the inflow depth. The p value is the exceedance probability of the annual precipitation. This figure was generated using Microsoft Office Excel 2013.

According to the annual shifts of impact patterns, the whole period (2004~2015) is divided into three subperiods, i.e., 2004~ 2007, 2008~2013 and 2014~2015 (Fig. 5). During the first subperiod from 2004 to 2007, the impact patterns are mainly in Class 1 (SRF:SIM-MS-MI). The annual precipitation amounts range from 882 mm to 941 mm, which are close to that of normal year (p = 50%, 896 mm). The main function of the Chaishitan Reservoir is deduced to discharge the stored water and flatten outflow in the whole year for downstream residential needs and agricultural irrigations^10,22,33^. The function probably belongs to the “run-of-river reservoir”^10,33^, and is also approximately similar to the regulation categories of headstream reservoirs in the nonflood season in the Huai River Basin, China^22^. During the second subperiod from 2008 to 2013, the main impact patterns are in Class 3 (SRF:EIMN-HS-MI). The annual precipitation amounts range from 527 mm to 960 mm, with the mean of 720 mm being close to that of dry year (p = 75%, 697 mm). The regulation mainly increases the flow magnitude and its variability in the pre-nonflood season to relieve the severe drying of the downstream rivers^10,33^. The main function is also the frequent discharge of the stored water in the nonflood season for downstream water consumption, particularly for the agricultural irrigations in the spring. The regulation is also similar to that of the “run-of-river reservoir”^10,33^ but is strengthened in the flow magnitude in the pre-nonflood season and the flow stability compared to the metric variations of Class 1. During the last subperiod from 2014 to 2015, the main impact patterns are in Class 4 (MRF:MDM-SS-HI). The annual precipitation amounts range from 938 mm to 1137 mm, which are close to or much greater than that of wet year (p = 25%, 940 mm). The regulation mainly decreases the flow magnitude in the flood season, but increases the flow magnitude in the nonflood season, thereby resulting in more stable flow with lower frequency and longer durations of both the high and low flow events^2,10,33^. This function is generally accepted as a conventional water quantity regulation for most reservoirs in the world, i.e., storing water and reducing the peak flow in the flood season for the safety of flood control (“storage reservoir”), while discharging water in the nonflood season for downstream water consumption (“run-of-rive reservoir”)^2^.

Therefore, because of the annual variations in both upstream precipitation and inflow, the regulation of the Chaishitan Reservoir shows remarkable annual shifts to guarantee the downstream water consumption of residential needs and agricultural irrigations (e.g., Kunming city), and to ensure the safety of flood control. Compared to the inflow regime metrics, the outflow magnitudes are increased slightly in most years, particularly in the pre-nonflood season (January~March) and flood season (April~September), and are decreased at the moderate impact grade around 2014. The outflow variation is interchanged between variability and stability, and the flow event durations are extended whose increments are gradually shifted from the moderate grade to the slight grade. The impacts of outflow intermittency are changed from the slight grade to the high grade, and the Julian dates of both the annual maximum and minimum daily outflows are delayed, being shifted from the slight grade to the high grade.

## Discussion and Conclusions

This paper tests the hypothesis that the impact patterns of reservoir regulation are variable, rather than stable over time. The results of the case study of the Chaishitan Reservoir show that:

(1). The regulation of the Chaishitan Reservoir slightly or moderately increases the mean daily flow magnitude in the flood season of most years, and even obviously increases that in the nonflood season over time, particularly in the pre-nonflood season. These phenomena occur because Chaishitan is a multiyear regulating reservoir of large storage capacity, which is strongly advantageous for changing the interannual distributions of outflow, i.e., to store runoff in wet years (e.g., 2004, 2005) and release in dry years (e.g., 2010~2012). Thus, the daily flow regimes gradually become stable, except in the pre-nonflood season, and the frequencies of low flow events are decreased, whereas those of high flow events are increased, particularly for extreme high flow events at the extremely impact grade. Moreover, the flow event durations are slightly extended, and the mean rates of both positive and negative changes of flow events increased before 2011 and then decreased. However, the impacts on flow timing do not exhibit obvious temporal patterns during the entire period. The most impactful metrics are the mean daily runoff magnitude in the pre-nonflood season (January-March), the frequencies of low flow events and extreme low flow events, the zero-flow days and the Colwell’s Constancy of mean daily runoff.

(2) Four annual impact patterns are identified for the regulation of the Chaishitan Reservoirs and obviously annual shifts are detected in the whole period, which are divided into three subperiods. The regulation is gradually enhanced with typical features of different hydrological years in a comprehensive manner. In dry years, the main regulation characteristic is the slightly regulated flow regime with slightly discharging the stored water and reduction of the peak outflow in the whole year, moderate stability and moderate intermittency. In normal years, the main regulation characteristic is the slightly regulated flow regime with extremely increasing flow magnitude in the pre-nonflood season, high stability and slight intermittency. In wet years, the main regulation pattern is the moderately regulated flow regime with moderately decreasing the flow magnitude in the flood season but extremely increasing flow magnitude in the nonflood season, along with slight stability and high intermittency.

The regulation characteristics are closely related to the annual variations of upstream precipitation or inflow, operational objectives, and so forth. The annual shifts are typically used to ensure the downstream water requirements of both production and residence, as well as the safety of flood control. The main regulation of the reservoir still focuses on the water resource utilization, that causes the flow regime to be stable and intermittent, with more regulation-induced flow events of longer durations. All of the current regulations promote the local developments of agriculture and residence. However, the potential environmental consequences should be not ignored, e.g., sedimentation in the reservoir caused by the more stable flow, aquatic organisms experiencing changes from turbulent flows to quiescent water pools, and the diversity loss of the aquatic river system in the downstream caused by the high intermittency of low flow events (particularly the zero-flow days). The future regulation of the Chaishitan Reservoir should further consider the river ecosystem health and environmental deterioration to relieve its negative impacts. Some proposed regulation strategies include weakening the interannual regulation capacity, inducing more low flow events with short duration in the nonflood season, and increasing the peak flow in the flood season. Additionally, future studies should also be implemented to consider the responses of river ecosystem to flow alteration, multi-objective optimization for interannual regulations, reservoir ecosystem services evaluation and environmental flow assessment.

## Material and Methods

### Study area and data source

The Chaishitan Reservoir is located in the Nanpan River, upstream of the Pearl River in Yunnan Province, South China (Fig. 1). The construction of the Chaishitan Reservoir was started in 1997 and finished in 2002. The Chaishitan Reservoir is a multi-objective and multiyear regulation reservoir, that provides a drinking water supply, agricultural irrigation, flood control and power generation. The total storage capacity and active storage capacity are 4.37 × 10^8^ m^3^ and 3.40 × 10^8^ m^3^, respectively (Table 2). In the Chaishitan catchment, the total area is 4,469 km^2^ and the major land covers are forest (46.17%) and paddy (46.02%). The climate characteristic belongs to the temperate climate region with dry winter and warm summer, with an annual average precipitation and temperature of 778 mm and 21.4 °C, respectively. There are two inflow tributaries, i.e., the Maitian River and Nanpan River.

**Table 2:** General characteristics of the Chaishitan Reservoir as well as the inflow and outflow stations.

	Sub-catchments	Maitian	Tianshen	Chaishitan
Topography	Area (km^2^)	439.7	3786.7	4469
	Elevation (meters above sea level)	1959–2053	1845–2344	1845–2344
	Slope	0.25–0.30	0.18–0.32	0.18–0.32
Land use (%)	Paddy land	24.33	49.67	46.02
	Forest	75.12	42.21	46.17
	Grassland	0.00	4.33	4.43
	Water	0.12	1.06	1.03
	Urban	0.43	2.72	2.35
Weather	Precipitation (mm)	779.2	762.2	764.0
	Temperature (°C)	21.4	21.4	21.4
Reservoir capacity	Dead storage capacity (10^8^m^3^)	—	—	0.85
	Flood control capacity (10^8^m^3^)	—	—	3.40
	Active storage capacity (10^8^m^3^)	—	—	3.40
	Total storage capacity (10^8^m^3^)	—	—	4.37
	Regulation capacity (%)			41.56

Long-term observed inflow and outflow series are collected for our study. The daily runoff series from 2004 to 2015 are collected from the Maitian hydrological station in the Maitian tributary, the Tianshen hydrological station in the upper Nanpan River and the Chaishitan hydrological station in the reservoir downstream, respectively. The controlled areas of the Maitian and Tianshen stations are 439.7 km^2^ and 3,786.7 km^2^, the sum of which covers the vast majority of the Chaishitan catchment (94.6%). Therefore, the sum of the daily runoff series at the Maitian and Tianshen hydrological stations is considered to represent the inflow series of the Chaishitan Reservoir, and the runoff series at Chaishitan station is the outflow series. The inflow ranges from 0.00 m^3^/s to 666.19 m^3^/s (July 4^th^, 2015) with a mean of 33.33 m^3^/s, and the outflow ranges from 0.00 m^3^/s to 469.50 m^3^/s (August 26^th^, 2015) with a mean of 32.85 m^3^/s. The entire period also contains wet, normal and dry years.

## Methods

### Flow regime metrics

The entire flow regime is usually fully characterized by five basic components, including magnitude, variability and frequency, duration, timing, and rate of changes; these components have been widely used to characterize long-term hydrographs in a detailed manner^18^. Twenty-two metrics are selected in our study to cover all of the basic components, i.e., four magnitude metrics, eight variability and frequency metrics, five duration metrics, three timing metrics and two metrics regarding the rating of change^24,36^ (Table 3). All of these metrics are calculated annually based on flow observations and the continuous time series of flow regime metrics are finally formed for both inflow and outflow in the whole period. Three seasons are divided for each year, i.e., the pre-nonflood (January-March), post-nonflood (October-December) and flood (April-September) seasons. To assure the comparability of frequency and duration metrics between inflow and outflow events, the thresholds of high and low flow events are set as the same values for both inflow and outflow series. The thresholds of extremely high (10^th^ percentile: MDF10), high (25^th^ percentile: MDF25), low (75^th^ percentile: MDF75) and extremely low (90^th^ percentile: MDF90) flow events are determined by the flow duration curve of the inflow series because it is not affected by the Chaishitan Reservoir. The corresponding values are 72.74 m^3^/s for MDF10, 36.04 m^3^/s for MDF25, 7.10 m^3^/s for MDF75 and 3.39 m^3^/s for MDF90.

**Table 3:** Flow regime metrics used for description of long-term hydrograph.

No	Groups	Flow regimes	Hydrologic Metrics	Abbreviation	Unit
1	Magnitude	average flow	Mean daily runoff	MDF	m^3^s^−1^
2			Mean daily runoff in the flood season (April-September)	MDFF	m^3^s^−1^
3			Mean daily runoff in the pre-nonflood season (January-March)	MDFN1	m^3^s^−1^
4			Mean daily runoff in the post-nonflood season (October-December)	MDFN2	m^3^s^−1^
5	Variability and Frequency	average flow	Coefficient of Variation (CV) of the mean daily runoff	CVDF	none
6			CV of mean daily runoff in the flood season	CVDFF	none
7			CV of mean daily runoff in the pre-nonflood season	CVDFN1	none
8			CV of mean daily runoff in the post-nonflood season	CVDFN2	none
9		low flow	Low flow spell count (75th percentile of MDF)	FDF75	none
10			Extremely low flow spell count (90th percentile of MDF)	FDF90	none
11		high flow	High flow spell count (25th percentile of MDF)	FDF25	none
12			Extremely high flow spell count (10th percentile of MDF)	FDF10	none
13	Duration	low flow	Low flow spell duration	DDF75	days
14			Extremely low flow spell duration	DDF90	days
15			Number of zero-flow days	Dzero	days
16		high flow	High flow spell duration	DDF25	days
17			Extremely high flow spell duration	DDF10	days
18	Timing	average flow	Colwell’s Constancy of the mean daily runoff	TDFC	none
19		low flow	Julian date of the annual minimum daily runoff	TMnDF	none
20		high flow	Julian date of the annual maximum daily runoff	TMxDF	none
21	Rating	average flow	Mean rate of positive changes in flow from one day to the next	RRise	none
22			Mean rate of negative changes in flow from one day to the next	RFall	none

### Impact assessment of individual metrics

The discrepancies between inflow and outflow metrics are adopted to assess the impacts of reservoir regulation. The relative deviation is used for the impact assessments of magnitude, frequency and variability, rating of change, and the absolute deviation is used for the impact assessments of duration and timing. The equations are given as follows.

1 $$\{\begin{array}{c}A{D}_{i}={V}_{outflow,i}-{V}_{inflow,i}\\ R{D}_{i}=A{D}_{i}/{V}_{inflow,i}\times 100 \% \end{array},$$

where *RD*_*i*_ and *AD*_*i*_ are the relative and absolute deviations of the *i*^th^ metric, respectively; *V*_*outflow,i*_ and *V*_*inflow,i*_ are the outflow and inflow values of the *i*^th^ metric, respectively. If the deviation is greater than 0.0, then the dam regulation positively impacts the flow regime metric, whereas if it is less than 0.0, then the regulation negatively impacts the flow regime metric; when the deviation is equal to 0.0, the regulation has no impacts. To clearly present the regulation impacts, the deviation is further categorized into five impact grades based on percentiles or days:no (i.e., −5% ≤ AD ≤ 5%, −3 days ≤ RD ≤ 3 days), slight (i.e., −25% ≤ AD < −5% or 5% < AD ≤ 25%, −15 days ≤ RD < −3 days or 3 days < RD ≤ 15 days), moderate (i.e., −50% ≤ AD < −25% or 25% < AD ≤ 50%, −30 days ≤ RD < −15 days or 15 days < RD ≤ 30 days), high (i.e., −75% ≤ AD < −50% or 50% < AD ≤ 75%, –91 days ≤ RD < −30 days or 30 days < RD ≤ 91 days) and extreme (i.e., AD < −75% or AD > 75%, RD < −91 days or RD > 91 days)^22,34^.

### Dimensionality reduction

High dimensionality and multicollinearity are key obstacles to flow regime analysis when a large number of metrics are used. As classical statistical methods, principal component analysis has strong advantages in merging numerous correlated metrics into a few independent composite components without losing metric information, and cluster analysis is quite competitive in identifying some typical characteristics from numerous chaotic information^19,34^.

In this study, the annual impact grades of all flow regime metrics are applied to further identify the impact patterns of reservoir regulation on the entire flow regimes, and their annual shifts. Principal component analysis is adopted to reduce the multicollinearity among all 22 flow regime metrics. If the threshold of cumulative variance of all metrics is no less than 75% of the total variance, then the first *m* principal factors are selected for further analysis. Subsequently, cluster analysis is adopted to cluster the impact grades of 12 years into some temporally representative variations. Therefore, the impact patterns of dam regulation on the entire flow regimes are identified, and the annual shifts are the alternate variations between different impact patterns over time. The Euclidean distance^37^ is used to calculate the similarity of the principal components between different years, and then Ward’s method^38^ is used for hierarchical clustering of the principal component series. Moreover, the Goodman–Kruskal index (*GKI*)^39^, the C index (*CI*)^40^, and the minimum cluster size are used to assess the cluster performance and determine the final class number. The most robust clustering is determined by the greatest value of *GKI* and the lowest value of *CI*^22^. All class sizes should be no less than two to ensure the representativeness and integrity of the individual classes.

Both principal component analysis and cluster analysis are implemented in the R platform (version 3.1.1)^41^; specifically, they are based on the princomp function in the stats package^41^, the hcluster function in the amap package^42^ and the cluster.stats function in the fpc package^43^.
